# Drug-nanoencapsulated PLGA microspheres prepared by emulsion electrospray with controlled release behavior

**DOI:** 10.1093/rb/rbw033

**Published:** 2016-09-21

**Authors:** Shenglian Yao, Huiying Liu, Shukui Yu, Yuanyuan Li, Xiumei Wang, Luning Wang

**Affiliations:** ^1^School of Materials Science and Engineering, University of Science and Technology Beijing, Beijing 100083, China,; ^2^State Key Laboratory of New Ceramics and Fine Processing, School of Materials Science and Engineering, Tsinghua University, Beijing 100084, China,; ^3^Department of Oral and Maxillofacial Surgery, School of Stomatology, Dalian Medical University, Dalian 116044, China,; ^4^Institute for Neuroscience, Capital Medical University, Beijing 100069, China

**Keywords:** PLGA microspheres, drug nanoencapsulation, emulsion electrospray, controlled drug release

## Abstract

The development of modern therapeutics has raised the requirement for controlled drug delivery system which is able to efficiently encapsulate bioactive agents and achieve their release at a desired rate satisfying the need of the practical system. In this study, two kind of aqueous model drugs with different molecule weight, Congo red and albumin from bovine serum (BSA) were nano-encapsulated in poly (dl-lactic-co-glycolic acid) (PLGA) microspheres by emulsion electrospray. In the preparation process, the aqueous phase of drugs was added into the PLGA chloroform solution to form the emulsion solution. The emulsion was then electrosprayed to fabricate drug-nanoencapsulated PLGA microspheres. The morphology of the PLGA microspheres was affected by the volume ratio of aqueous drug phase and organic PLGA phase (*V_w_/V_o_*) and the molecule weight of model drugs. Confocal laser scanning microcopy showed the nanodroplets of drug phase were scattered in the PLGA microspheres homogenously with different distribution patterns related to *V_w_/V_o_*. With the increase of the volume ratio of aqueous drug phase, the number of nanodroplets increased forming continuous phase gradually that could accelerate drug release rate. Moreover, BSA showed a slower release rate from PLGA microspheres comparing to Congo red, which indicated the drug release rate could be affected by not only *V_w_/V_o_* but also the molecule weight of model drug. In brief, the PLGA microspheres prepared using emulsion electrospray provided an efficient and simple system to achieve controlled drug release at a desired rate satisfying the need of the practices.

## Introduction

Controlled-release strategies allowed for drug delivery in a controllable pattern at a specific location and time present numerous advantages over conventional drug administration, such as prolonged therapeutic period, enhanced efficacy, reduced systemic toxicities, eliminated dosage and improved patient compliance [1–3]. Polymeric drug delivery systems (DDS) based on biocompatible and biodegradable polymers are extensively used in recent for the controlled release of single or multiple therapeutic agents in the treatments of many diseases like injured tissue regeneration and targeted cancer therapy owing to their specific advantages [4, 5]. Polymeric DDS are easy to be fabricated, chemically modified and adjusted degradation rate and achieve long-term sustained drug release via physical encapsulation or chemical immobilization. And most importantly, they do not need surgical removal upon complete drug release. Therefore, many types of polymeric DDS such as microspheres, nanoparticles, lipospheres and micelles attract increasing attentions as drug carriers for the release of proteins, genes, antigens, peptides or chemicals. 

Injectable biodegradable polymer microspheres based on poly (dl-lactic-co-glycolic acid) (PLGA) are thought to be a very simple system to obtain ideal controlled release profiles [6, 7]. For example, Jiang *et al.* developed a kind of PLGA microspheres for the release of vaccine antigens, which could be easily injected through a syringe needle with minimal discomfort and provide long-term release of vaccine antigens and improved immunogenic responses in mammalian subjects relative to soluble antigen [8–11]. Besides, PLGA is a Food and Drug Administration (FDA)-approved biocompatible copolymer that has been extensively used in biomedical devices with excellent application records *in vivo*. Despite these benefits, a number of challenges still remain with the development of PLGA microspheres.

One of the primary issues is to attain well-controlled drug release profiles in a simply way that should match with the demands of diseases. For example, long-term steady administration of drugs may be beneficial for the treatment of many diseases such as cancer, rheumatoid arthritis or viral disease in individuals with compromised immune system [12, 13]. While for some kinds of diseases like diabetes, insulin as one of the treatment drugs to manage the blood glucose, needs to be quickly and effectively delivered [14, 15]. Another key issue is the instability of loaded drug during encapsulation process. There are various types of methods developed for the fabrication of polymeric drug-released microspheres, such as emulsion, emulsion-solvent evaporation, coacervation and spray drying methods. The water-in-oil-in-water double emulsion method has been widely used to prepare PLGA microspheres because of the simplicity of the fabrication processes and the ability to generate large and reproducible output [16]. However, PLGA microspheres prepared using the double emulsion technique frequently resulted in low encapsulation efficiency for water-soluble macromolecules, and loss of bioactivity [17, 18]. For most of these methods, the harsh conditions such as shear or cavitation forces, excessive heat, freezing and drying involved in these encapsulation methods often cause significant inactivation of drugs like vaccine antigens [19, 20]. For the emulsion electrospray method, electric field is added to the drug emulsion solution to spray particles. And compared with the traditional techniques, it has the potential to reduce denaturation of protein drugs and afford tighter regulation over particle size distribution and morphology [21–23].

In this work, water-dissolved drugs were nano-encapsulated into PLGA microspheres by emulsion electrospray method, and the released behavior of the drug-loaded PLGA microspheres was investigated. Two kinds of model drugs, Congo red with 696.98 Da of molecular weight (M_w_) and albumin from bovine serum (BSA) with 68 kDa M_w_, were dissolved into deionized water to form the aqueous drug phases. And then PLGA microspheres were prepared by electrospraying the emulsion solution. Scanning electron microscope (SEM) and laser scanning confocal microscope (LSCM) were used to characterize the morphology and drug phase distribution within the PLGA microspheres. The release behavior was tested *in vitro* and the proposed mechanism for the release of water-dissolved drugs from the PLGA microspheres was summarized.

## Materials and methods

### Materials

Poly (dl-lactide-co-glycolide), PLGA (M_w_ 50 000 Da) ether terminated, with a 75/25 ratio (PLA/PGA) from Jinan Daigang Biomaterial Co., Ltd (Shandong, China), was used as biodegradable polymer. Congo red (C_32_H_22_N_6_Na_2_O_6_S_2_, Biotopped Beijing, China) and BSA (albumin from bovine serum, Sigma) were used as model drugs to research the water-soluble molecule released from the PLGA microsphere. BSA-FITC (fluorescein conjugated BSA) was used to research the drug distribution. All chemicals were used directly without further purification.

### Fabrication of PLGA microspheres

**Figure 1.**
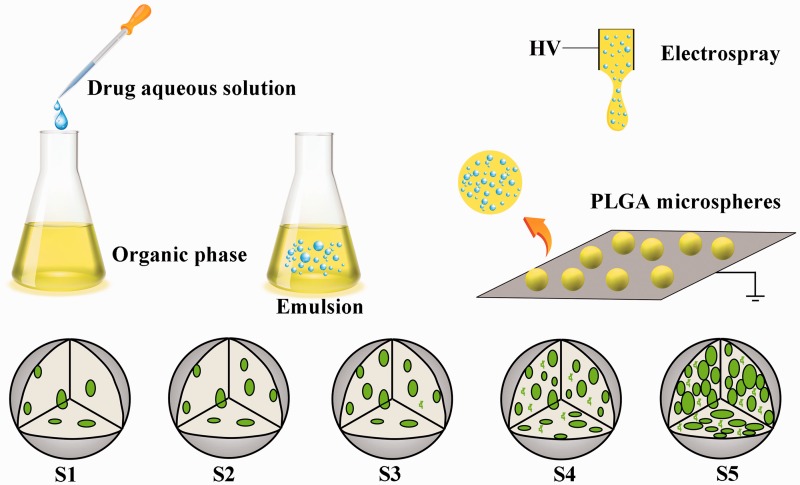
Schematic representation of the emulsion electrospray process. Aqueous drug phase solution was added into the organic phase (PLGA chloroform solution), and then the mixture was sonicated to form emulsion solution for electrospray. S1–S5 indicated the different distribution of aqueous nanodroplets in the PLGA microspheres with different *V_w_/V_o_*.

Firstly, 6 wt% PLGA solution as the organic phase was prepared by dissolving appropriate amounts of PLGA copolymer in chloroform. And the aqueous phase solution was prepared by dissolving Congo red or BSA in deionized water. Then, a mixture of aqueous phase and PLGA solution was sonicated (5 cycles, 10 s, 40 W) to form emulsion solution at various volume fractions. And the solutions were then immediately electrosprayed (<1 h) with a flow rate of 1 mL/h and a high voltage (HV) of 6 kV supplied to the needle. The PLGA microspheres were collected with an aluminum foil with a distance of 20 cm. The schematic diagram of the fabrication process was shown in Fig. 1. From S1 to S5, the *V_w_/V_o_* were 5, 10, 20, 50 and 100 μL/mL, respectively.

### Characterization

The aluminum foil with collected PLGA microspheres was cut into 1 × 1 cm and then sputter-coated with Au after drying in air for 24 h. And then, the samples were imaged using a JEOL JSM-7001F field-emission SEM at an accelerating voltage of 10 kV. The average particle size of the microspheres was analysed by Image Pro Plus, and at least 250 microspheres were measured for each sample. To study the distribution of drug in the PLGA microspheres, BSA-FITC-loaded microspheres were observed by LSCM (Zeiss 780) without drying process. The encapsulation efficiencies (EE) were calculated according to the following equation [12]:

(1) $$EE=\frac{\;\text{Total}\;\text{observed}\;\text{amount}\;\text{of}\;\text{Congo}\;\text{red}\;\text{in}\;\text{PLGA}\;\text{microspheres}}{\text{Total}\;\text{amount}\;\text{of}\;\text{Congo}\;\text{red}\;\text{loading}}\times 100\%.$$

### Drug release profiles

PLGA microspheres (0.2 g) were added into PE tube vial containing 2 mL of phosphate-buffered saline (PBS) release buffer. At least three repeats for each sample group were conducted. The tubes were then mounted on a Labquake Rotisserie with 60 rad/min at 37°C, and 1 mL of the release buffer was collected through centrifuge and replaced by 1 mL of fresh buffer at each predetermined time point. To investigate the release profile of encapsulated Congo red inside the microspheres, absorption of Congo red under 485 nm (characteristic adsorption wavelength of the Congo red in PBS) was measured using a UV-vis spectrophotometer (Beijing Rayleigh Analytical Instrument Co., China) at room temperature. The Quartz sample cell was sealed up, and the absorption spectra under 485 nm were recorded at a same time interval with the reference spectrum of PBS. And the BSA concentration was obtained by BCA protein assay kit (Beyotime) via multimodal plate reader (PerkinElmer, USA). The drug released rate was calculated by the formula (2):

(2) $$E=\frac{V_{E}\sum_{1}^{n-1}C_{i}+V_{0}C_{n}}{m_{0}}\times 100\%,$$

where *E* is the release rate of drug at each collected time point, *V_0_*, *V_E_* and *m*_0_ are constants for 2 mL, 1 mL and 0.2 g, respectively in this work. *i* and *n* are the numbers of experiment time points. *C_i_* and *C_n_* are the concentration of drug at the *i* and *n* time point.

## Results and discussion

### Particle morphology and size distribution

**Figure 2.**
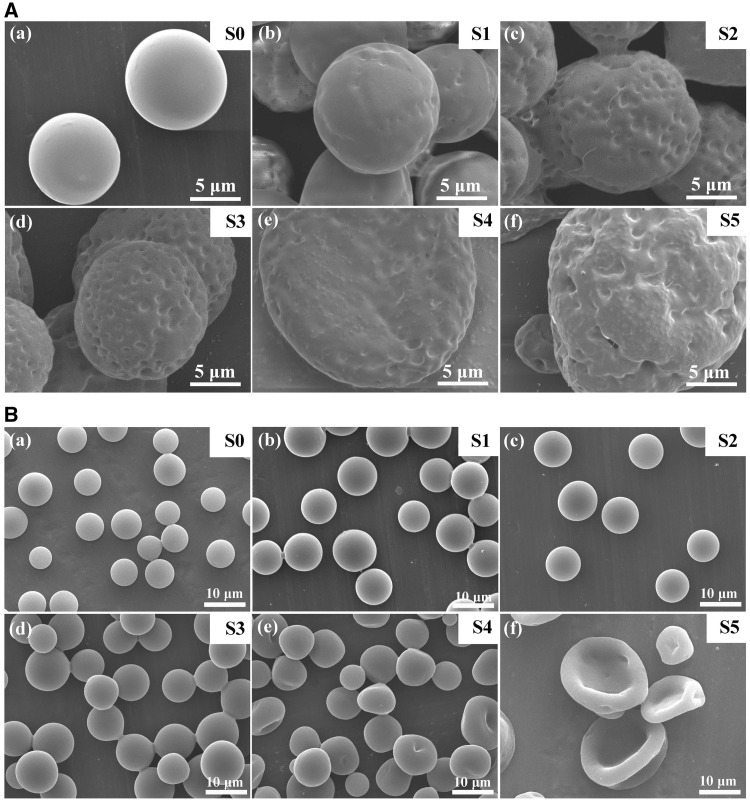
Typical morphologies of BSA-loaded PLGA microspheres prepared with different *V_w_/V_o_* of 0 (a, S0), 5 (b, S1), 10 (c, S2), 20 (d, S3), 50 (e, S4) and 100 μL/mL (f, S5). The BSA concentrations were 0.001 (**A**) and 0.4 g/mL (**B**), respectively.

The typical morphologies of the electrosprayed PLGA microspheres loaded with different volume of BSA solution (S0–S5, S0 represents pure PLGA microspheres without aqueous drug added) were shown in Fig. 2. The concentrations of BSA used in Fig. 2A and B were 0.001 and 0.4 g/mL, respectively. For the lower BSA concentration group (Fig. 2A), the pure PLGA microspheres were round and plump with smooth surfaces. With the involvement of aqueous phase solution, the surface of the microspheres became rougher because of the aqueous nanodroplets distributed on the surface. At the same time, the shape of the PLGA microspheres changed from spherical to irregular with the aqueous phase increased. For the higher BSA concentration group (Fig. 2B), the particles were round but had no obvious dimples on the surface of the microspheres when the aqueous phase volume was <50 μL for per milliliter organic phase. While, when the aqueous phase volume increased to 50 μL (S4), the particle shape was not perfectly spherical with depressed pits. And the microspheres turned to biconcave discs when the aqueous phase increased to 100 μL (S5). Therefore, it is obvious that the aqueous volume ratio has effect on the morphology of PLGA microspheres. The more the aqueous phase, the less the sphericity of PLGA microspheres.

Comparing the morphologies of the two groups, lower concentration of BSA could more easily lead to more pits on the surface. In a diluted aqueous phase, because more water existed in the nanodroplets trapped within the surface and interior of spheres, their evaporation during drying left more empty spaces, resulting in more pits. More than that, for both concentrations of BSA solution, when the aqueous phase volume is more than 50 μL, the shape of the PLGA microspheres started to become irregular with various shapes. During the electrospray process, the PLGA microspheres loaded with aqueous phase nanodroplets were sprayed form the syringe needle to the grounded copper plate. And in the process, both of the water and organic solvent should sufficiently evaporate from the microspheres before falling down to the collection plate. While, for the volatility of water is quite lower than that of chloroform, the solvent of the PLGA microspheres with more aqueous phase could not totally evaporate before falling down to the copper plate. And the landing of the semi-solidified PLGA microspheres on the copper plate under the electric field force and gravity could affect the shape and sphericity of the microspheres. Furthermore, with the further evaporation of solvent in air, the surface of the PLGA microspheres collapsed and led to rough surfaces. The morphologies of Congo red-loaded PLGA microspheres were similar and not shown here.

**Figure 3.**
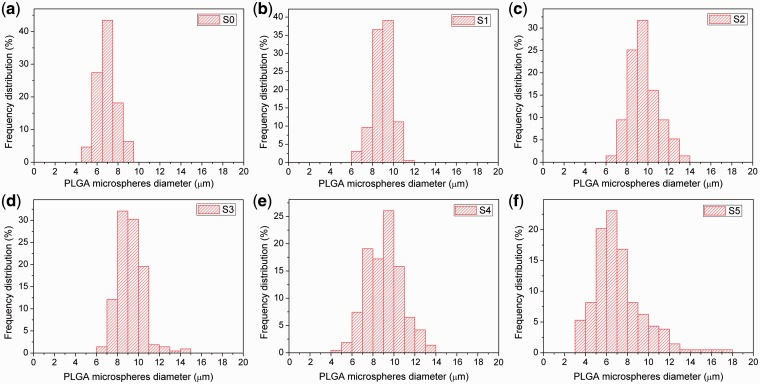
Size distribution of PLGA microspheres fabricated with *V_w_/V_o_* of 0 (**a**, S0), 5 (**b**, S1), 10 (**c**, S2), 20 (**d**, S3), 50 (**e**, S4) and 100 μL/mL (**f**, S5).

The size of PLGA microspheres could be adjusted by the electrospray parameters. In order to get appropriate drug release profile and implement direct injection, around ten micrometer of microspheres were fabricated. As shown in Figs 2 and 3, the particle diameter did not change in a large range. The average particle diameter of PLGA microspheres were measured to be about 6.9 ± 1.0, 8.9 ± 0.9, 9.6 ± 1.4, 9.2 ± 1.2, 9.1 ± 1.7 and 7.2 ± 2.4 μm for S0–S5, respectively. Figure 3 showed the distribution of microspheres diameter. When the volume of aqueous phase was <100 μL, the diameters of PLGA microspheres (S1–S4) were similar. When the volume was added to 100 μL, the electrosprayed PLGA microspheres (S5) were not quite uniform and the mean diameter of particles reduced because the microspheres collapsed during the drying process. However, the range of the particle size distribution was increased with the increase of *V_w_/V_o_*. Especially for the S5 condition, the microspheres had the broadest size distribution that was probably because of the increased conductivity of the electrospray solution with more aqueous phase.

The encapsulation efficacy and mean particle size were summarized in Table 1. Comparing to traditional methods, the encapsulation efficacy of the PLGA microspheres prepared by the emulsion electrospray method were over 80% that is benefit for the drug delivery applications [12, 22]. In this work, with the aqueous phase volume ratio increased, the EE decreased from 92 to 80%. For higher aqueous phase volume could increase the density of emulsion droplets in the electrospray solution, therefore more aqueous drug would locate on the surface of microsphere that could cause drug loss during the collection process, and that reduced the final encapsulation efficacy.

**Table 1.** Characterization of the emulsion electrospray PLGA microspheres

Types	*V_w_/V_o_*	Particle shape	Mean particle diameter (μm)	Encapsulation efficacy
S0	0	Perfectly spherical	6.9 ± 1.0	0
S1	5 μL/mL	Perfectly spherical	8.9 ± 0.9	92%
S2	10 μL/mL	Perfectly spherical	9.6 ± 1.4	90%
S3	20 μL/mL	Spherical	9.2 ± 1.2	86%
S4	50 μL/mL	Not perfectly spherical	9.1 ± 1.7	81%
S5	100 μL/mL	Various shapes	7.2 ± 2.4	80%

### Drug distribution within the PLGA microspheres

**Figure 4.**
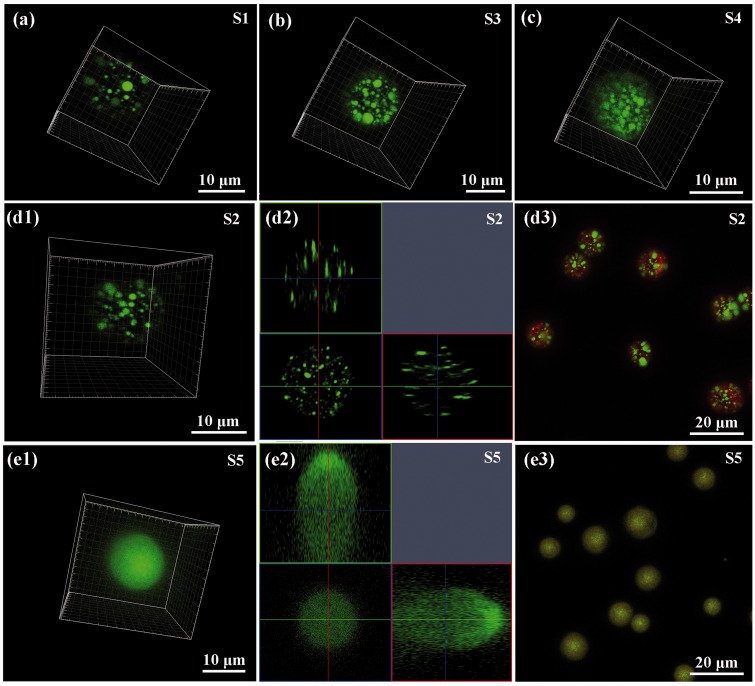
The BSA-FITC distribution in the PLGA microspheres with different *V_w_/V_o_* of 5 (**a**, S1), 10 (d1–d3, S2), 20 (**b**, S3), 50 (**c**, S4) and 100 μL/mL (e1–e3, S5). (a–c), (**d1**) and (**e1**) are the 3D reconstruction of z-stack confocal images. (**d2**) and (**e2**) are the cross-section of PLGA microspheres; (**d3**) and (**e3**) are the drug phase (BSA-FITC was green) distribution in the PLGA microspheres (red was rhodamine). Full color version available online.

In order to investigate the drug distribution within the PLGA microspheres, fluorescence-labeled BSA, BSA-FITC was used to visualize the encapsulated drug under the fluorescence microscopy. And the fluorescence dye of rhodamine was also added to the PLGA chloroform solution to indicate the microsphere outline. The BSA distributions within the different formulation of PLGA microspheres were shown in Fig. 4. The 3D reconstructions of LSCM images for BSA-FITC (Fig. 4(a–c), (d1–d3) and (e1–e3)) confirmed that more drugs were encapsulated with more aqueous solution added. And the green dots indicated the formation of nanodroplets of aqueous phase solution, which dispersed over the PLGA emulsion homogenously by sonication. For the S1–S4 PLGA microspheres with the aqueous phase volume <100 μL for per milliliter organic phase, the distribution of drug phase was discrete. For the S5 PLGA microsphere, the BSA nanodroplets aggregated forming continuous aqueous phase. 3D drug distribution detail was shown in supplementary movie 1. The results indicated that the nanodroplets of model drug formed in the emulsification process could be maintained in the subsequent electrospray. And the density of the drug nanodroplets was increased with the volume ratio of the aqueous phase. At the same time, with the increase of *V_w_/V_o_*, BSA-FITC in aqueous nanodroplets could diffuse into the PLGA molecules entanglements to make the interface of aqueous/organic phases indistinctly. Especially when the volume ratio increased to 100 μL/mL (Fig. 4(e1–e3)), BSA-FITC dispersed in PLGA microspheres uniformly. This may be caused by two main reasons: one reason was the diffusion and intrusion of the BSA-FITC molecules into the PLGA phage when the solvent evaporated from the microspheres; the other reason was the aggregation of the drug phase nanodroplets forming continuous distribution. And this was also proved by the SEM results in Fig. 6(e1), which showed the pits at the nanoscale on the surfaces of PLGA microspheres caused by the dissolution of Congo red molecules were close and connected. Therefore, the LSCM and SEM showed the drug distribution in the PLGA microspheres was primarily determined by *V_w_/V_o_*.

### Drug release behavior

**Figure 5.**
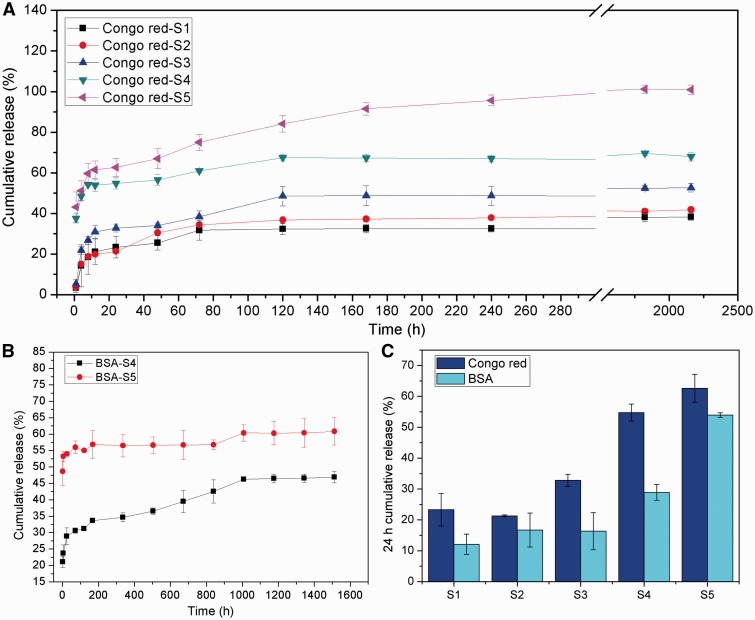
Release profiles of Congo red (**A**) and BSA (**B**) from PLGA microspheres in PBS (pH = 7.0) at 37 °C (**C**) showed the cumulative release percentage of Congo red and BSA in the first 24 h.

**Figure 6.**
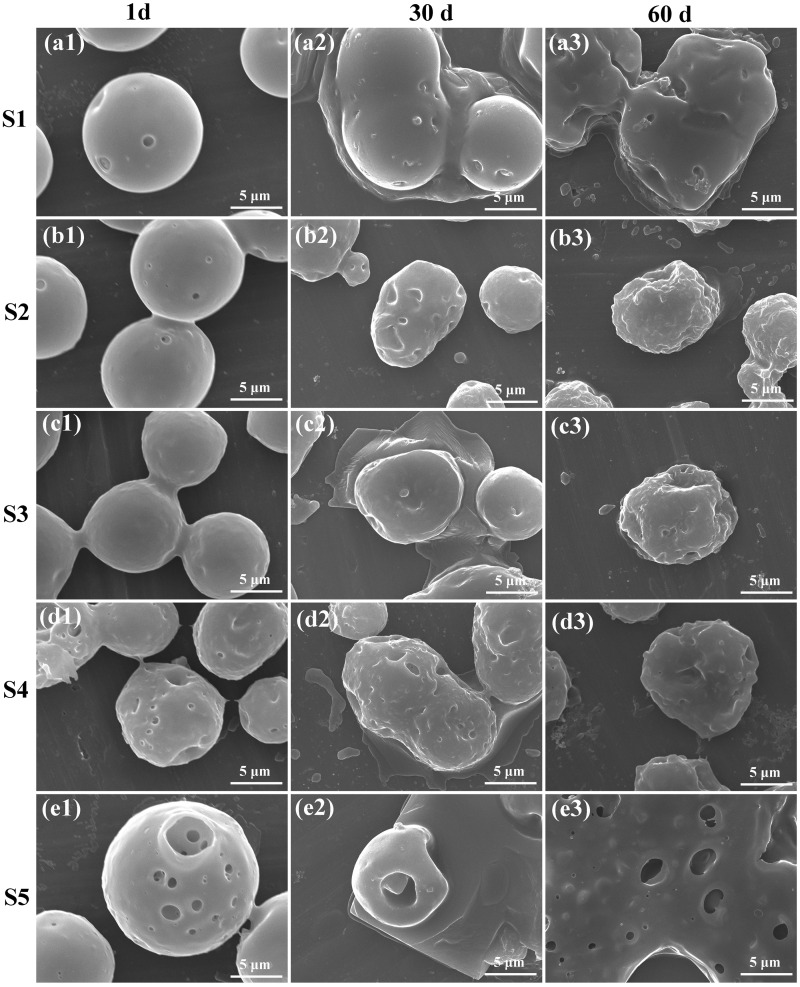
Typical morphology of PLGA microspheres with Congo red released after 1, 30 and 60 days of incubation *V_w_/V_o_* were 5 (**a1**–**a3**), 10 (**b1**–**b3**), 20 (**c1**–**c3**), 50 (**d1**–**d3**) and 100 μL/mL (**e1**–**e3**), respectively.

In order to investigate the drug release behaviors through the electrosprayed PLGA microspheres, Congo red and BSA were used representing small and large molecular drugs, respectively. The *in vitro* release behaviors of Congo red and BSA were displayed in Fig. 5. And the typical SEM morphologies of Congo red-loaded PLGA microspheres after different time of drug release were shown in Fig. 6. It could be clearly seen that both of the Congo red and BSA release behaviors were strongly influenced by *V_w_/V_o_*. Congo red and BSA had very similar release kinetic. Three typical stages could be concluded according to the rate of drug release.

The first stage is the initial burst release stage within the first 24 h, during which the drug released very quickly. The cumulative release percentages of BSA and Congo red within 24 h were summarized in Fig. 5C. It could be seen that the more the aqueous phase solution was added, the quicker the drug released from the PLGA microspheres. According to the drug distribution within the microspheres, it is obviously that the drug nanodroplets located on/near the surface shells of the PLGA microspheres would release out foremost [24]. Therefore, the PLGA microspheres with more drug solution added contained more drug nanodroplets located on/near the surface shells and apparently had higher drug release rate. Moreover, in the groups of Congo red S4, S5 or BSA S5, more than 50% of the drugs were released in this stage, which was much higher than other groups. This is probably due to the connectivity of drug nanodroplets in the microspheres with high *V_w_/V_o_*. Figure 6 showed the typical morphologies of PLGA microspheres after different time of *in vitro* drug release. The aqueous phase of drug nanodroplets on the surfaces of PLGA microsphere will dissolve in PBS solution immediately, leaving small pits and holes on the PLGA microspheres, as shown in Fig. 6(a1–e1). Furthermore, the release rate of Congo red was higher than that of BSA at the same volume ratio, which indicated the molecule weight also has effect on the drug release in this stage. And for the drug molecules in the surface shell but not exposed to the release buffer in the first 24 h, they could diffuse into PBS with the water penetration into the microspheres.

The second stage is a slow drug release stage with a stable release rate before PLGA degradation. During this stage, the drugs encapsulated inside the PLGA microspheres will release out slowly along with the diffusion and penetration of the PBS solution into the microspheres. For the Congo red S5 group (Fig. 5A), the cumulative release percentage approximately achieved to 100% in the initial 10 days. That is because of the formation of highly continuous aqueous phase in S5 group. It is much easier for PBS solution to diffuse into the PLGA microspheres through the continuous aqueous phase than PLGA phase. And once the encapsulated drugs in the surface shell exposed to the release buffer, the inner drugs could rapidly diffuse through the interconnected water channel. While for the S1–S4 groups, the Congo red molecules maintained released from the spheres until 2 month later, and the cumulative release percentage were 38, 41, 52 and 68%, respectively after 2 months of incubation. Fig. 6(a2–e2) showed the typical morphologies of the Congo red-released PLGA microspheres in the second stage.

After 30 days of incubation in the PBS buffer, PLGA molecules started to swell and degrade to desquamate from the surface of microspheres, and the desquamated PLGA pieces even dispersed into the release buffer that caused the deformation of microspheres. After 60 days of incubation, PLGA microsphere S5 disintegrated, leaving a flat PLGA film with holes only, while for the S1–S4 groups, the PLGA microspheres developed to shrink particles with rugged surfaces. The BSA-loaded PLGA microspheres had similar release behavior with Congo red (Fig. 5B), but the release rate was slower and the cumulative release percentage was less in comparison with Congo red S4 and S5 groups because of the notable difference of molecule weights of BSA and Congo red.

### Mechanism on drug release from PLGA microsphere

**Figure 7.**
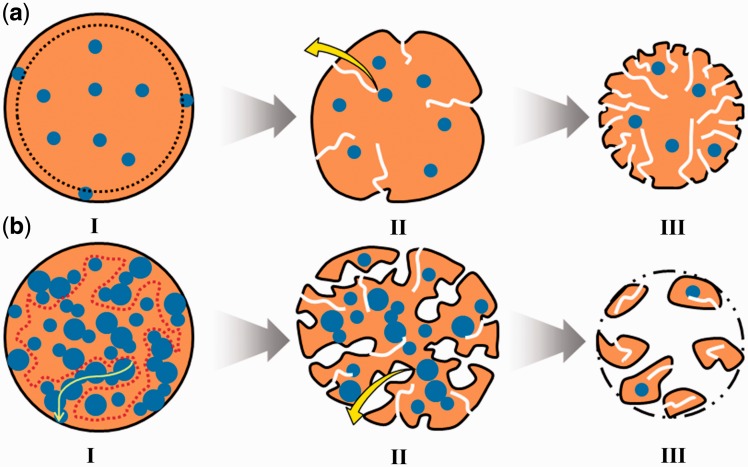
Schematic illustration of the proposed mechanism for the release of water-dissolved drugs from the PLGA microspheres. (**a**) and (**b**) show the degradation and drug released process of S1 and S5, and the process of others conditions is similar and between them. State I: initial microspheres before degradation, drugs in the surface layer or connected to the surface could quickly release in the first 24 h. State II and III: water penetrated into the microspheres and PLGA molecule peeled off from the microspheres surface, the drug released slowly from the microspheres through diffusion controlled.

Drug-encapsulated biodegradable polymers attract more and more attentions for the drug controlled delivery of pharmacological agents to their targets at a therapeutically optimal rate and dosage. In this work, the drug release behavior of the PLGA microspheres prepared by the emulsion electrospray could be easily tailored by changing the aqueous/organic volume ratio. To better understand the drug release behaviors through the PLGA microspheres fabricated with different aqueous/organic volume ratio, a possible mechanism was proposed in Fig. 7. During the initial period of incubation, the shape of microspheres maintained and the drugs in the surface layer or connected to the surface quickly released. With the increase of *V_w_/V_o_*, the connectivity of drug phase in the microspheres was improved, and that could promote the drug molecule diffuse through the connective channels that was showed as the curve arrow in Fig. 7(b) state II. In the following incubation in PBS, water penetrated into the microspheres and PLGA molecule peeled off from the microspheres surface, the drug released slowly from the microspheres through diffusion controlled [25, 26]. Similarly, high connectivity of the drug phase also could promote the drug molecule diffusion. Therefore, the drug release behavior could be regulated by changing the parameter of aqueous/organic volume ratio. Especially for multiply drugs release, our method provides a simple tool to controlled release different drugs by adjusting the aqueous/organic volume ratio in terms of their different molecular weights (S. Yu, S. Yao, Y. Wen, submitted for publication).

## Conclusion

This study developed a simple and effective way to nano-encapsulate water-dissolved drugs in PLGA microspheres in a gentle condition. Comparing to the traditional emulsion method, the emulsion electrospray showed higher drug encapsulation efficiency and the easily controlled drug release behavior by simply changing the volume ratio of aqueous phase and organic phase. With the increase of *V_w_/V_o_* from 0 to 100 μL/mL, the aqueous drug nanodroplets in the microsphere became connected that caused the different drug release behaviors. Therefore, the PLGA microspheres prepared using emulsion electrospray provided an efficient and simple system to achieve controlled drug release at a desired rate satisfying the need of the practices.

## Supplementary Material

supplementary movie 1
