# Supplementary material for: Drug-nanoencapsulated PLGA microspheres prepared by emulsion electrospray with controlled release behavior
Source: Regen Biomater. 2016 Sep 21;3(5):309–17. doi: 10.1093/rb/rbw033 (PMC5043157; doi:10.1093/rb/rbw033)
Supplement: supplementary movie 1 [file rb_rbw033_index.html]

Supplementary Data | Regenerative Biomaterials

## Supplementary Data

files

- Supplementary Data - zip file
